# Mucosa-associated lymphoid tissue lymphoma of the lacrimal gland: A case report and literature review

**DOI:** 10.1097/MD.0000000000038303

**Published:** 2024-05-24

**Authors:** Qin Zhong, Yu Yan, ShuangLe Li

**Affiliations:** a Department of Ophthalmology, Zigong First People’s hospital, Zigong, Sichuan Province, China.

**Keywords:** case report, lacrimal gland, mucosa-associated lymphoid tissue lymphoma, ocular adnexal lymphoma

## Abstract

**Rationale:**

The most common subtype of primary lymphoma of the ocular adnexa is the mucosa-associated lymphoid tissue (MALT) subtype. MALT lymphoma of the lacrimal gland is relatively rare among the lacrimal gland tumors, and the early clinical symptoms are atypical, which can easily lead to misdiagnosis and missed diagnosis. Here, we report a case of MALT lymphoma of the lacrimal gland and explore its clinical manifestations, pathological characteristics, management, and pathogenesis, with the aim of helping clinicians gain an in-depth understanding of ocular adnexal MALT lymphoma.

**Patient concerns:**

A 60-year-old man presented to our hospital with proptosis and diplopia. The right eye deviated and shifted toward the lower part of the nose.

**Diagnosis:**

Orbital enhanced magnetic resonance imaging suggested a mass with a maximum cross-section of 3.2 × 2.1 cm. T1 weighted image was isointense, and the enhancement was more uniform and obvious.

**Interventions:**

The right orbital mass was treated surgically, and the final pathology report was MALT lymphoma. After the pathological report was released, the patient was transferred to the hematology department for further diagnosis and no further treatment was given eventually.

**Outcomes:**

Seven months later, the patient did not complain of discomfort. Whole-body positron emission tomography-computed tomography, superficial lymph node examination and orbital magnetic resonance imaging revealed no abnormal changes.

**Lessons:**

The clinical manifestations of MALT lymphoma are heterogeneous. Imaging examination is important for assessing the size of the tumor and its relationship with adjacent tissues. Postoperative pathological examination may provide further evidence for the evaluation of the patient’s surgical efficacy and prognosis. Management of MALT lymphoma of the lacrimal gland requires a multidisciplinary approach involving ophthalmologists, hematologists, and radiotherapists.

## 1. Introduction

The concept of mucosa-associated lymphoid tissue lymphoma (MALT lymphoma) was first proposed in 1983 by Isaacson and Wright in their study of *Helicobacter pylori*. It was first found in the digestive tract, but was later found to occur in the lungs, skin, thyroid and ocular appendages.^[1]^ The revised European-American classification of lymphoid neoplasms (REAL) proposed by the International Lymphoma Study Group in 1994, formally distinguishes mucosa-associated lymphoid tissue-type lymphomas of the ocular appendages as a type of marginal zone B-cell lymphoma. In 2001, the World Health Organization (WHO) further improved the REAL classification, but the classification related to lymphoma of the ocular adnexa was not significantly changed, and the 2 were basically the same, which was categorized as extranodal marginal zone B-cell lymphoma of mucosa-associated lymphoid tissue lymphomas, referred to as MALT lymphoma.

In recent years, the incidence of lymphoma has increased annually. Some researchers have reported that the annual incidence rate of ocular adnexal lymphoma has increased by as much as 4.5%.^[2]^ The most common subtype of primary lymphoma of the ocular adnexa is the MALT lymphoid tissue subtype. MALT lymphoma of the lacrimal gland is relatively rare among the lacrimal gland tumors. Although most ocular adnexal MALT lymphomas have a relatively good prognosis, the clinical manifestations of mucosa-associated lymphoid tissue lymphoma are highly variable, lack specificity, and are difficult to diagnose, leading to confusion among clinicians in their diagnostic work. MALT lymphoma is rarely symptomatic in the early stage of the disease. Consequently, there is often a delay in ophthalmic consultations and diagnosis. Therefore, it is particularly important to study related issues in ocular adnexal lymphoma. In this study, we report a case of mucosa-associated lymphoid tissue lymphoma of the lacrimal gland and review its clinical manifestations, pathology, management, and prognosis, with the aim of helping clinicians gain an in-depth understanding of ocular adnexal MALT lymphoma.

## 2. Case report

**Figure 1.**
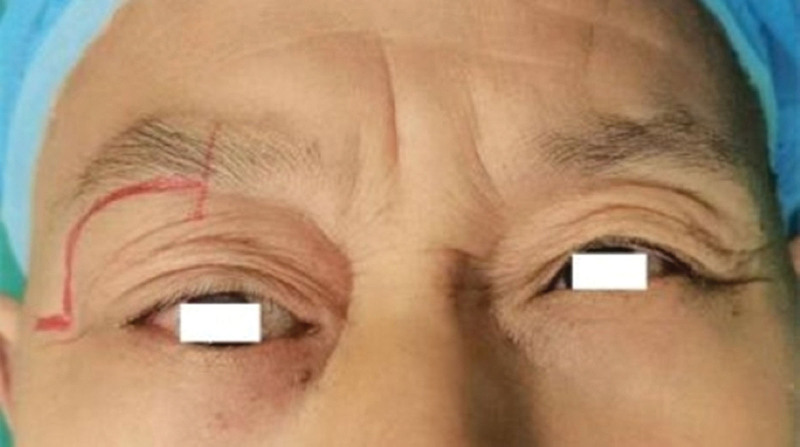
The patient has presented with protrusion of the right eye. The right eye was deviated and shifted toward the lower part of the nose.

**Figure 2.**
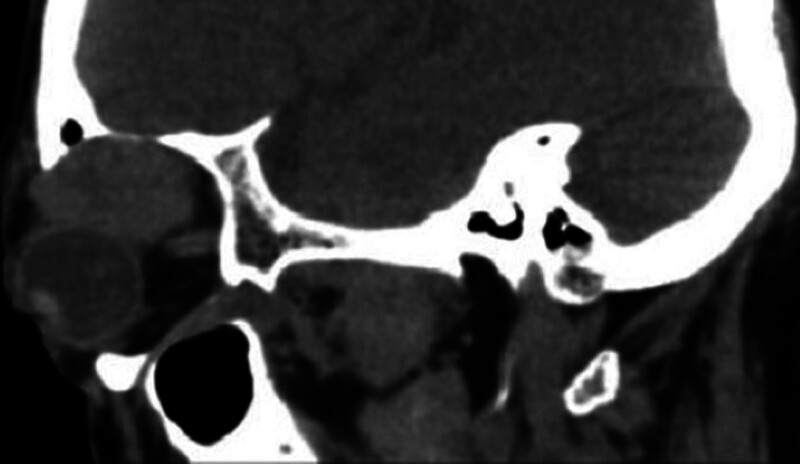
CT scan suggested an oval soft tissue mass (approximately 3.3 × 2.3 × 2.4cm in size, with clear boundary, uniform density, enlarged lacrimal fossa, and no bone destruction) in the extrapyramidal space. CT = computed tomography.

**Figure 3.**
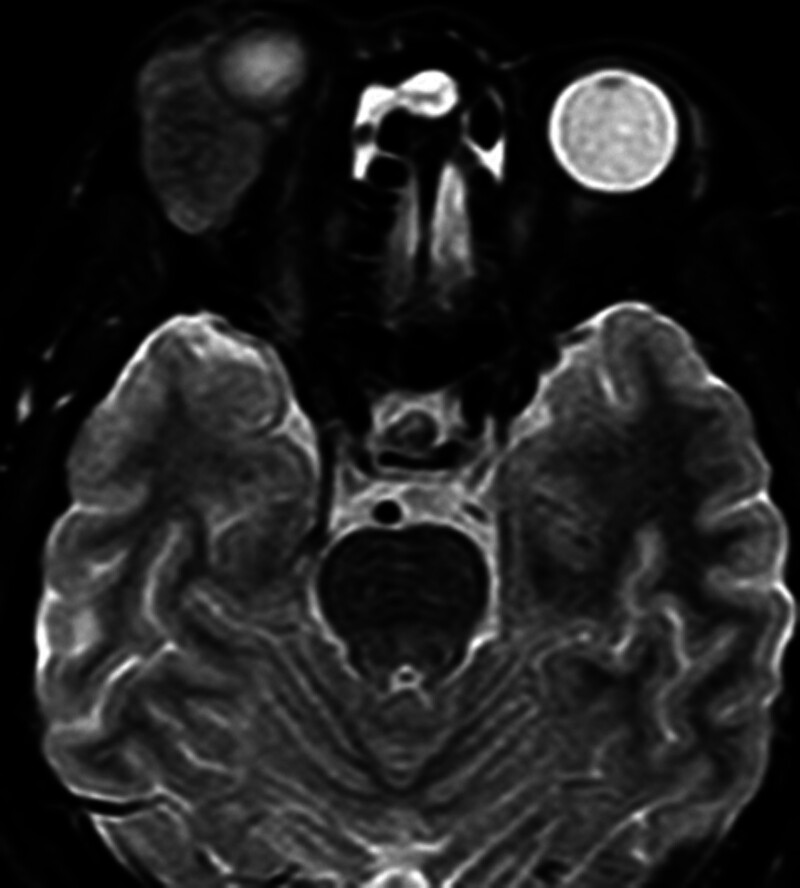
MRI suggested that the right upper quadrant of the orbit (outside the muscle cone) had an abnormal signal. T1WI was isosignal, and the enhancement was more uniform and obvious. MRI = magnetic resonance imaging, T1WI = T1 weighted image.

A 60-year-old man (Fig. 1) complained of proptosis and diplopia for 2 years, and admitted to our hospital as “right orbital mass” in May 2023. He was not given any interventions prior to the clinic visit. He had a 5-year history of chronic obstructive pulmonary disease and asthma, without a history of trauma or surgery. Since 2021, the patient has presented with protrusion of the right eye without any causative triggers, accompanied by diplopia, no vision loss, and no systemic symptoms. The exophthalmos of both eyes was 17 and 12 mm, respectively. A moderately hard mass approximately 3 × 1 cm in size can be palpated at the upper margin of the right eye, with poor mobility, and no obvious pulsation, accompanied by slight pressure pain. The right eye was deviated and shifted toward the lower part of the nose, and there was a significant restriction of the right eye’s outward and upward movement, while the movement of the left eye was normal. B-ultrasound suggested a solid supraorbital mass in the right eye, with blood flow signals visible within the mass, and no abnormalities in the superficial lymph nodes of the head and neck. Computed tomography (CT) scan of the orbit (Fig. 2) suggested an oval soft tissue mass (approximately 3.3 × 2.3 × 2.4 cm in size, with clear boundary, uniform density, enlarged lacrimal fossa, and no bone destruction) in the extrapyramidal space, meanwhile, chest and head CT showed no abnormality. Orbital enhanced magnetic resonance imaging (MRI) (Fig. 3) suggested that the right upper quadrant of the orbit (outside the muscle cone) had an abnormal signal, with a uniform signal and a maximum cross-section of 3.2 × 2.1 cm. T1 weighted image (T1WI) was isosignal, and the enhancement was more uniform and obvious. The adjacent extraocular muscles and bone were compressed, and there were no obvious signs of bone destruction; therefore, neoplastic changes were considered.

**Figure 4.**
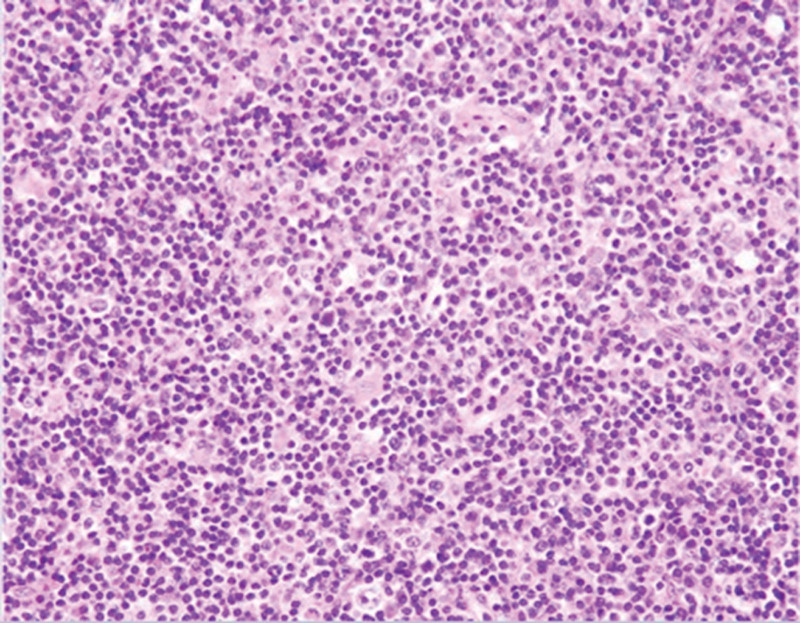
HE × 300.

The patient underwent surgical treatment under general anesthesia, and the operation was successful with good postoperative recovery. Our pathology department considered the mass as a lymphoma (Fig. 4) and sent the pathology section to the Remote Consultation Center for further staging. The final pathology report was MALT lymphoma. The pathology report suggested a nodular back-to-back distribution of microscopically segregated fibrous lesions in the tumor, with a germinal center visible within the lesion. Abundant plasma cell infiltration is observed near of the fibrous septum.

Immunohistochemical staining showed that the germinal centers were CD20+, CD79a+, PAX5+, CD10+, BCL2−, and BCL6+. The Ki67 positivity rate was approximately 60%. Plasma cells were CD79a+, PAX5+, EMA+, MUM1+, CD138+, and CD38+ with Kappa+ greater than Lambda+, and the Ki67 positive rate was approximately 15%. The CD21+ FDC network was expanded. Background small lymphocytes were CD3+ and CD45RO+. CD30 expression in individual cells was positive, except for ALK, CD15, EBV, and CK which were negative. Combined with the above pathology reports, the pathologist considered the tumor to be a MALT lymphoma, which could be further confirmed by IGH and IGK gene rearrangement monitoring.

After the pathological report was released, the patient was transferred to the hematology department for further diagnosis and treatment. The hematologist did not provide any treatment after a series of examinations, but advised him to undergo regular follow-up in the ophthalmology and hematology departments. The patient’s test results, including the Chlamydia psittaciosis nucleic acid test, thyroid function, Epstein–Barr virus and antinuclear antibody, were negative. The results of T-lymphocyte subsets suggested that the absolute values of lymphocytes was 1646.0 × 10^6^/L, the absolute value of total T-lymphocytes were 1320.0 × 10^6^/L, and the absolute value of CD3+ CD4+ cells were 784.0 × 10^6^/L. Bone marrow flow cytology did not reveal any significant abnormal cells. A bone marrow smear showed active myeloproliferative activity, with 7% mature lymphocytes and no obvious abnormal cells.

The patient was very satisfied with the results of the treatment and presented a banner as a token of appreciation. Three months after surgery, the patient was reviewed in the ophthalmology clinic, with no complaints of any physical discomfort. Simultaneously, a whole-body positron emission tomography-CT was performed with no abnormal results. Seven months later, the patient did not complain of discomfort. Superficial lymph nodes and orbital MRI revealed no abnormalities.

Our study was exempted from review, and this exemption complied with the policies of our institutional review board.

## 3. Discussion

Marginal zone lymphoma (MZL) is a B-cell lymphoma originating in the marginal zone. They are classified into 3 subtypes according to their site of origin, namely extranodal MZL(EMZL), lymph node MZL, and splenic marginal zone MZL. Most EMZLs arise in the mucosal tissues; hence, these lymphomas are also defined as MALT lymphomas. MALT lymphoma usually displays an indolent course, and its prognosis is better than that of splenic MZL and lymph node MZL.^[3–5]^ The most common subtype of primary lymphoma of the ocular adnexa is the MALT subtype, accounting for approximately 67% to 80% of cases, accounting for around 8% of all non-Hodgkin lymphomas.^[4,6,7]^ The next most common lymphoma types in the ocular adnexa region are follicular, mantle-cell, and diffuse large B-cell lymphomas, each accounting for approximately 10% of primary ocular adnexal MALT lymphoma.^[7]^

### 3.1. Clinical types and manifestations

Retrospective analyses of ocular adnexal lymphoma have revealed some variability in the distribution among different subtypes, which may be related to ethnicity and geography.^[4]^ Ocular adnexal MALT lymphoma is mainly primary and can also be secondary to lymphoma in other systems, with diffuse or localized nodular growth.^[8,9]^ Ocular adnexal MALT lymphoma most commonly affects the anterior part of the orbit and the lacrimal gland. In addition, it can also affect the eyelids, conjunctiva, lacrimal sac, and extraocular muscles.^[3]^ Clinical types are categorized according to the primary site of the lesion and the extent of imaging lesions involved, and can be distributed into orbital, lacrimal, eyelid, and conjunctival types. Ocular adnexal MALT lymphomas are more frequent unilaterally, and their clinical manifestations vary depending on the site of the lesion.^[3]^ Orbital MALT lymphoma is often located in the supraorbital region, and often manifests as exophthalmos, ocular dislocation, and ocular motility disorders, which can be accompanied by vision loss and diplopia in some patients. It can be found in the supraorbital region with a tough mass, unclear boundary, and corresponding eyelid swelling. The eyeball often shifts inward and downward. Our case presented mainly with proptosis, diplopia and significant limitation of upward and outward eye rotation, similar to previous reports. Eyelid-type MALT lymphoma often presents with painless eyelid swelling, which can lead to ptosis in severe cases; early manifestations of conjunctival lymphoma include congestion and edema of the conjunctiva, rough conjunctiva, and a “fish egg like” protrusion. As the tumor increases, the conjunctival mass gradually increases and merges into a piece, presenting a “fish like” appearance that can be palpated on the eyelids. The destruction of conjunctival goblet cells leads to a decrease in tear quality and causes dry eye in patients.

### 3.2. Diagnostic imaging methods

MALT lymphoma is a space-occupying lesion that can be diffusely infiltrative or well-demarcated. Except for conjunctival MALT lymphoma, where the extent of the lesion can be seen directly, most patients require diagnostic imaging to clarify the extent of the lesion. Commonly used diagnostic imaging methods include ultrasound, CT, and MRI. MALT lymphoma is mostly a hypoechoic lesion on B-mode ultrasound, and in some patients the internal echoes may be heterogeneous. Color Doppler ultrasonography can show the blood flow within the tumor and its relationship with the surrounding tissues.

CT has qualitative diagnostic value for orbital bone structure and the relationship between the extent of tumor invasion and the surrounding tissues. MRI is the gold standard for diagnosing ocular adnexal lymphoma, with a high resolution for soft tissue. MRI can reflect the location of the lesion and the involvement of surrounding structures, can differentiate between other tumors and tumor-like lesions, and has a higher sensitivity to lesion involvement of the sclera than other imaging tests. Orbital MALT lymphoma is often characterized by poorly defined prostrate casting-like changes on imaging, and may also encircle the eyeball, spreading to both the inside and outside of the muscle cone, but less often causing lesions of the orbital wall. Enhanced MRI revealed significant enhancement of the lesion. Lacrimal gland MALT lymphoma is characterized by inflammatory enlargement of the lacrimal gland, the scope of which is mostly bounded by the lateral rectus and superior rectus muscles, and rarely breaks through the internal space of the muscle cone. The lesion shows an “amygdaloidal” change in the axial position, and a “crescentic or hemicrescentic” change in the coronal position. In our case, orbital enhanced MRI suggested that the right upper quadrant of the orbit (outside the muscle cone) had an abnormal signal. T1WI was isointense, and the enhancement was more uniform and obvious; the adjacent extraocular muscles and bone were compressed, and there were no obvious signs of bone destruction. The imaging presentation of the mass was similar to those reported in previous studies. Imaging manifestations of eyelid-type MALT lymphoma mostly reveal diffuse thickening of the eyelids, and striated and mass lesions. Conjunctival MALT lymphoma of the limited type is characterized by localized thickening of the eyelids and conjunctiva, with the lesion adhering to the sclera in front of the eye, which may be triangular or “teardrop shaped” in the sagittal plane. MALT lymphoma of the extraocular muscle can be seen on imaging as a full-length diffuse hypertrophy of the involved extraocular muscles. Oculomotor MALT lymphoma can be seen in all extraocular muscles, but the superior rectus and levator palpebral muscles are most commonly involved.

### 3.3. Pathogenesis and risk factors

The pathogenesis of MALT lymphoma has been shown to be associated with chronic antigenic stimulation, and these antigens include microbial pathogens,^[10]^ as well as autoimmune antibodies.^[11]^ MALT lymphoma develops from mature B lymphocytes that have been triggered by antigens for prolonged periods. Lymphomas are often preceded by inflammatory precursor lesions. Self-antigens or infectious antigens may act as persistent antigenic triggers that lead to a disease. Sustained antigenic stimulation of B cells by specific bacterial infections or self-antigens underlies the pathogenesis of MALT lymphoma.^[5]^ Sustained antigenic stimulation drives an aberrant immune response, a process that involves the overactivation of B and T cells and the participation of other inflammatory cells and factors, stimulating abnormalities in related genes such as the NF-κB signaling pathway, which ultimately leads to genetic and molecular abnormalities such as chromosomal translocations and mutations, mutations, proliferation, and neoplastic transformation of B cells.^[12]^ The main factor in the development of this lymphoma is the constitutive activation of the NF-κB pathway, which occurs through various types of genetic alterations.^[5,13]^

There is substantial evidence that MALT lymphoma arises from a synergistic drive between oncogenic products and immune stimulation, which is associated with chronic pathogen infections or autophagic processes.^[5,12,14]^ Indeed, many studies have confirmed the relationship between the transformation of chronic inflammation and malignancy. *H pylori*-associated gastric MALT lymphoma is a classic model of infectious inflammation that promotes the transformation of inflammatory cancers.^[15]^ Stimulation by *H pylori*-associated antigens in gastric tissues attracts neutrophils to the site of inflammation and triggers sustained B-cell proliferation. The release of reactive oxygen species from these neutrophils can lead to a variety of genetic variants, and sustained B-cell proliferation also increases the risk for the development of gene mutations and genetic abnormalities. *H pylori* also activates the NF-κB pathway, which is a key regulator of the pro-inflammatory response, further suggesting a role of inflammation in MALT lymphoma.^[15]^

*Chlamydia psittaci* infection induces an antigen-driven process that may be a predisposing factor for ocular adnexal lymphomas with a prevalence of more than 50% in Italy and Korea.^[16]^ In our case, the Chlamydia psittacosis nucleic acid test was negative, so we did not consider the patient to have Chlamydia psittacosis. In addition to infectious factors, chronic inflammation due to autoimmune diseases such as rheumatoid arthritis, Hashimoto thyroiditis, and Sjogren syndrome can increase the risk of MALT lymphoma.^[12]^ A classic example of autoimmune disease-associated MALT lymphoma is salivary gland MALT lymphoma occurring in the setting of primary Sjogren syndrome.^[17]^

### 3.4. Treatment

Currently, there are no clear expert guidelines or consensus regarding the diagnosis and treatment of ocular adnexal MALT lymphoma. It is generally believed that the histopathologic subtype and clinical staging of the disease are the best predictors of prognosis and choice of treatment. Surgery is one of the mainstays of treatment for ocular adnexal MALT lymphoma, not only to remove the lesion, but also to obtain a specimen of the lesion for pathologic characterization to develop the next step in the treatment plan. Radiotherapy is the treatment of choice for isolated low-grade lymphoma, radiotherapy is a treatment of choice. For disseminated and high-grade lymphomas, chemotherapy, with or without radiotherapy, should be the treatment of choice.^[4]^ For ocular adnexal MALT lymphomas, local control, disease-free survival, and overall survival are good with radiation therapy. The results of treatment of non-MALT lymphomas using radiotherapy were also good, but not as favorable as the treatment results for MALT lymphomas.^[3,18]^

### 3.5. Prognosis

Most ocular adnexal MALT lymphomas have a relatively good prognosis; however, a few MALT lymphomas have relapsed and spread, and some have even transformed to high-grade aggressive lymphomas, among which patients with local tumor recurrence, extraorbital spread, and high-grade transformation have a poorer prognosis.^[19,20]^ Some researchers have reported that a higher frequency of peripheral blood Tregs and Treg/Th17 ratio might be associated with a favorable outcome in lymphoma patients, better response to chemotherapy, and a lower rate of relapse.^[21]^ Imaging examination of ocular adnexal MALT lymphoma has certain characteristics; therefore, it is important for primary diagnosis with a combination of clinical and imaging appearances to improve the preoperative diagnostic rate.

Some researchers have suggested that it is important to perform orbital imaging urgently in all patients with lacrimal gland tumors to obtain a provisional diagnosis.^[22]^ Most non-epithelial lacrimal gland tumors, including lymphoma, temporarily improve with systemic steroids,^[4,18]^ which is the cause of delayed diagnosis and possibly affects histologic diagnosis.^[23]^ Clinical manifestations are heterogeneous, and its management requires a multidisciplinary approach involving ophthalmologists, hematologists, and radiotherapists.

Our study has several limitations. Preoperatively, according to the patient’s clinical manifestations and imaging finding, our physician team considered the tumor to be a benign epithelial tumor of the lacrimal gland. Postoperatively, the pathology department considered the patient to have a lymphoma, and invited the pathology department of West China Hospital of Sichuan University to consult the patient’s case, and the final pathology report was MALT lymphoma of the lacrimal gland. We realize that our ability to predict the disease needs to be further improved, and we should learn more about the imaging of the tumor and collaborate and communicate with the radiology department preoperatively to obtain more accurate imaging features. This case suggests that immunohistochemistry is particularly important when the patient’s clinical presentation and imaging findings are atypical. Meanwhile, IGH and IGK gene rearrangement tests were not performed in our case to further confirm the diagnosis, and we need to develop a more comprehensive laboratory workup in the future. However, long-term results of pathologically confirmed cases of MALT lymphomas need further study because occasional relapses at distant sites can Occur. In addition, our current follow-up observation of the patient was only 7 months, which is too short, and we will continue to follow-up and observe the changes in the patient’s condition in the future.

In recent years, fluorescence in situ hybridization (FISH) technology and gene detection technology have been used to detect the molecular genetic features of MALT lymphoma, and the diagnosis of MALT lymphoma, which is now one of the most important means of lymphoma diagnosis internationally.^[24–26]^ To provide patients with more accurate diagnosis and treatment, we believe that a comprehensive examination is necessary. Owing to the limitations of the technology level, such technologies are not yet available routinely. In the future, researchers may need to invest more effort into enhancing the level of testing for the benefit of patients.

## 4. Conclusions

To conclude, the clinical manifestations of mucosa-associated lymphoid tissue lymphoma are heterogeneous, and the early clinical symptoms are atypical. Consequently, there is often a delay in ophthalmic consultations and diagnosis. This study reports a complete case of MALT and summarizes the clinical features of MALT lymphoma to help clinicians have a deeper understanding of the disease. The diagnosis of MALT lymphoma is not only based on clinical presentation, imaging features and histopathologic examination, but also needs to be combined with immunohistochemical detection methods. Localized MALT lymphoma of the lacrimal gland has a good prognosis, but a few patients may relapse locally or progress to high-grade lymphoma or systemic lymphoma, and long-term systematic follow-up of patients with lymphoma is recommended.

## Author contributions

**Data curation:** Qin Zhong, Yu Yan.

**Writing – original draft:** Qin Zhong.

**Writing – review & editing:** Qin Zhong.

**Conceptualization:** Yu Yan, ShuangLe Li.

**Methodology:** Yu Yan, ShuangLe Li.

**Supervision:** ShuangLe Li.
